# Mitochondrial regulation in the tumor microenvironment: targeting mitochondria for immunotherapy

**DOI:** 10.3389/fimmu.2024.1453886

**Published:** 2024-10-11

**Authors:** Minseo Ahn, Akhtar Ali, Jae Ho Seo

**Affiliations:** ^1^ Department of Biochemistry, Wonkwang University School of Medicine, Iksan, Republic of Korea; ^2^ Sarcopenia Total Solution Center, Wonkwang University School of Medicine, Iksan, Republic of Korea; ^3^ Institute of Wonkwang Medical Science, Wonkwang University, Iksan, Republic of Korea

**Keywords:** mitochondria, TME, immunotherapy, metabolism, immune evasion

## Abstract

Mitochondrial regulation plays a crucial role in cancer immunity in the tumor microenvironment (TME). Infiltrating immune cells, including T cells, natural killer (NK) cells, and macrophages, undergo mitochondrial metabolic reprogramming to survive the harsh conditions of the TME and enhance their antitumor activity. On the other hand, immunosuppressive cells like myeloid-derived suppressor cells (MDSCs), regulatory T cells (Tregs), mast cells, and tumor-associated macrophages (TAMs) rely on mitochondrial regulation to maintain their function as well. Additionally, mitochondrial regulation of cancer cells facilitates immune evasion and even hijacks mitochondria from immune cells to enhance their function. Recent studies suggest that targeting mitochondria can synergistically reduce cancer progression, especially when combined with traditional cancer therapies and immune checkpoint inhibitors. Many mitochondrial-targeting drugs are currently in clinical trials and have the potential to enhance the efficacy of immunotherapy. This mini review highlights the critical role of mitochondrial regulation in cancer immunity and provides lists of mitochondrial targeting drugs that have potential to enhance the efficacy of cancer immunotherapy.

## 1 Introduction

The tumor microenvironment (TME) is a complex and dynamic environment that plays a crucial role in cancer development, progression, and therapeutic response (1). From the perspective of cancer immunity, the TME is not only occupied by cancer cells but also consists of a variety of cell types, including both immunosuppressive cells and immune cells (2). Among the many factors within this environment, mitochondria stand out as critical regulators, not just serving as the powerhouse of the cell but also playing key roles in metabolic pathways, apoptosis, and cellular differentiation (3–5). These mitochondrial functions can significantly impact the behavior of cells within the TME. Immunotherapy has emerged as a new type of anticancer treatment that aims to strengthen immune cell function to fight cancer. Immune checkpoint inhibitors that block immune checkpoints, such as CTLA-4, PD-1, and PD-L1, are the most widely used immunotherapy. However, immune checkpoint inhibitors are not always effective because some cancer cells do not respond to the treatments (6, 7). Many studies have shown that mitochondrial-targeting drugs synergistically enhance the efficacy of immune checkpoint inhibitors while improving immune cell function and reducing the activity of immunosuppressive cells. Therefore, understanding the mechanisms of mitochondrial metabolic reprogramming is crucial for developing strategies to enhance immune cell function in the tumor microenvironment, which could lead to more effective cancer treatments through immunotherapy. In this mini-review, we briefly explain the role of mitochondrial regulation in cancer immunity within the TME and mitochondrial-targeting drugs that might serve as novel immunotherapeutic treatments.

## 2 Mitochondrial metabolic reprogramming in immune cells of tumor microenvironment

Mitochondrial metabolic reprogramming refers to the process by which the function and metabolic pathways of mitochondria are altered, often in response to changes in the cell’s environment or state (8). Within the TME, stress conditions, such as reduced oxygen consumption, elevated reactive oxygen species (ROS) generation, depolarized membrane potential, and impaired biogenesis disrupt the mitochondrial metabolism of immune cells and impair their function (9–11). Therefore, mitochondrial metabolic reprogramming plays a key role in the antitumor activities of immune cells that infiltrate the TME (12).

T cells are among the most important immune cells that are responsible for identifying and destroying cancer cells. When T cells are activated, their metabolism shifts from oxidative phosphorylation (OXPHOS) to glycolysis, supporting their rapid proliferation and effector functions (13). However, T cells show a persistent loss of mitochondrial function and mass when infiltrating tumors (14). Furthermore, continuous T cell stimulation in hypoxic environments leads to Blimp-1-mediated suppression of PGC-1α-dependent mitochondrial reprogramming (14). Several studies showed that enhancing mitochondrial metabolism improves T cell function. For instance, overexpression of PGC-1α enhanced mitochondrial biogenesis and metabolic capacity, thus improving CD8+ T cell antitumor effects (15). Stimulating T cell surface receptor 4-1BB enhances mitochondrial fusion and biogenesis in CD8+ tumor-infiltrating lymphocytes, independently of PGC-1α and p38-MAPK signaling (16).

Natural killer (NK) cells are crucial cytotoxic lymphocytes that are involved in the innate immune response against infected or transformed cells (17). In their inactive state, NK cells generate ATP primarily via mitochondrial OXPHOS, and upon activation, both glycolysis and OXPHOS increase, boosting ATP production (18, 19). A decreased level of PGC-1α, a master regulator of mitochondrial biogenesis, significantly impaired the ability of NK cells to control B16F10 melanoma growth *in vivo*, showing that mitochondria play a key role in NK cells (20). Upon infiltration of the TME, NK cells exhibit significant disadvantages. NK cells isolated from liver tumors exhibit fragmented mitochondria and impaired metabolism characterized by suppressed glycolysis and mitochondrial dysfunction (21). Furthermore, TGF-β induces metabolic dysfunction in NK cells from patients with metastatic breast cancer, leading to reduced glycolysis and OXPHOS and increased mitochondrial fragmentation (22). Blocking TGF-β and/or GARP can improve NK cell metabolism and function (22). NK cells showed a significant enhancement in immune activity and cytotoxicity when functional allogeneic mitochondria were transferred, indicating that mitochondrial improvement may restore NK cell function in the TME (23).

In the context of macrophage differentiation and function, mitochondria play a vital role in the regulation of metabolic reprogramming, signaling pathways, and immune responses (24). Among the two macrophage phenotypes, M1 and M2, M1 macrophages promote antitumor immunity by producing proinflammatory cytokines, such as IL-1β, IL-6, and IL-12 (25). M1 macrophages rely primarily on glycolysis for ATP production, favoring glycolysis even in the presence of oxygen, similar to the Warburg effect observed in cancer cells (25). Most macrophages in the TME exhibit the M2-like phenotype (25). This is due to the low glucose, high lactate levels, and hypoxic conditions of the TME, which drive macrophages to undergo M2 polarization (26, 27). Furthermore, tumors secrete cytokines and chemokines, such as IL-10, TGF-β, CCL2, and CSF-1, which recruit and polarize macrophages to the M2 phenotype (27).

## 3 Mitochondrial metabolic regulation in immunosuppressive cells

**Figure 1:**
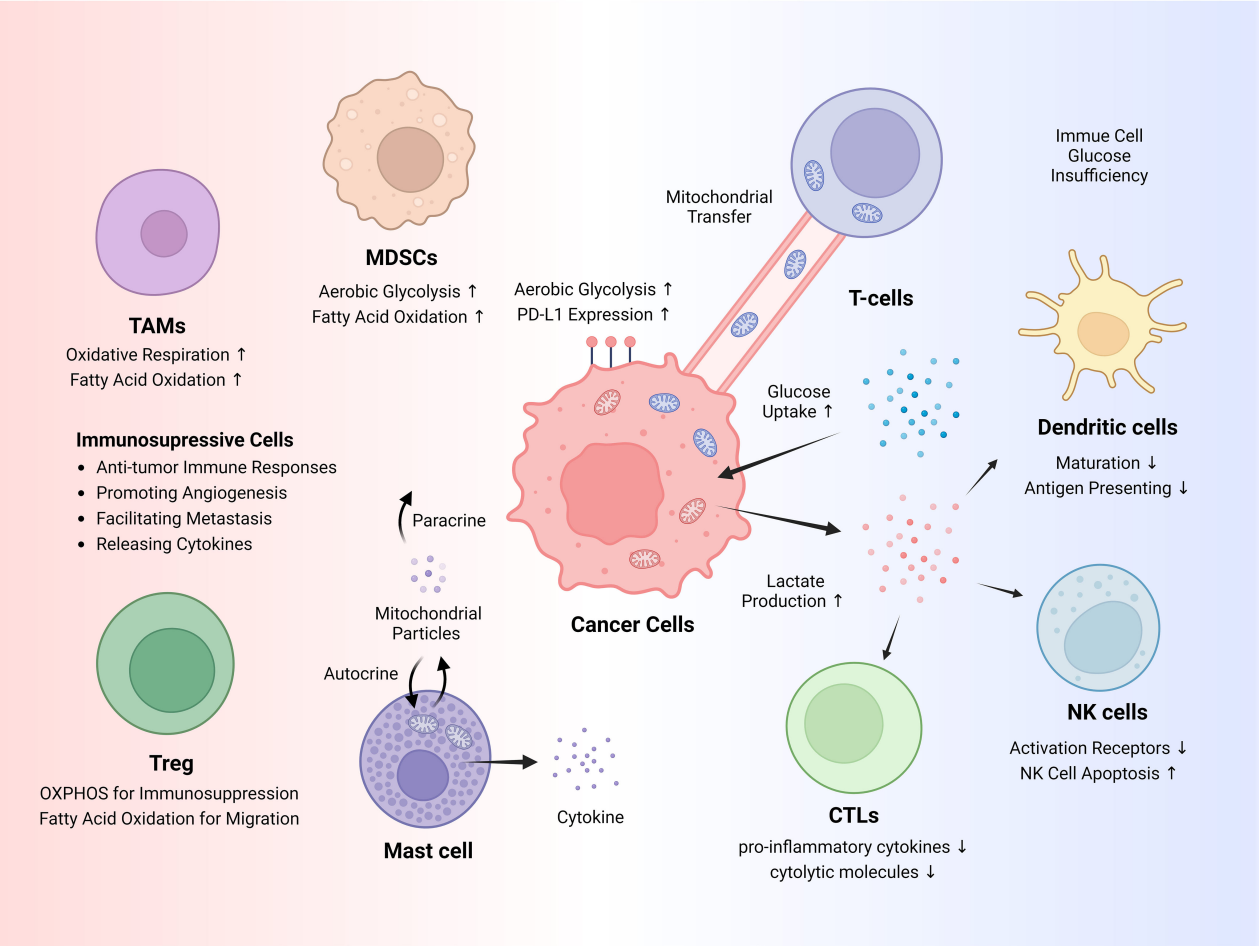
Cancer immune evasion and immunosuppressive cells. Cancer cells exhibit increased glucose uptake and heavily rely on glycolysis through a phenomenon known as the Warburg effect, a form of metabolic reprogramming. This heightened glucose uptake restricts glucose availability for immune cells, while lactate itself suppresses the antitumor activity of immune cells. Cancer cells can hijack mitochondria from T cells via mitochondrial transfer. Various immunosuppressive cells aid in tumorigenesis, and metabolic pathways, such as fatty acid oxidation (FAO) and oxidative phosphorylation (OXPHOS), adapt depending on the circumstances. NK, natural killer; Tregs, regulatory T cells; MDSCs, myeloid-derived suppressor cells; TAMs, tumor-associated macrophages.

The TME comprises diverse immunosuppressive cells that promote tumorigenesis and immune evasion (**Figure 1**). These include myeloid-derived suppressor cells (MDSCs), regulatory T cells (Tregs), mast cells, and tumor-associated macrophages (TAMs) (28, 29). Many studies have highlighted the essential roles of mitochondria and mitochondrial metabolism in immunosuppressive cells, indicating their potential as therapeutic targets.

MDSCs are immunosuppressive monocytes and neutrophils that rely heavily on glucose metabolism (30). For instance, β-adrenergic receptor signaling activates the STAT3 pathway, enhancing OXPHOS and glutamine utilization through the TCA cycle in MDSCs, reducing mitochondrial ROS via the NRF2 pathway, thereby improving MDSC survival (31). Decreased glucose availability, and consequently lactate production, results in smaller tumors, fewer MDSCs, and improved antitumor immune responses (32). Lipid metabolism is also crucial for MDSCs function. Tumor-derived cytokines activate STAT3 and STAT5 pathways and upregulate lipid transport receptors of MDSCs (33). This increases lipid uptake and enhances oxidative metabolism and immunosuppressive functions of MDSCs (33). Blocking fatty acid oxidation (FAO) in MDSCs, where fatty acid uptake and oxidation are crucial, reduces their immunosuppressive functions (34).

Tregs are another type of immunosuppressive cell that suppress the function of various immune cells, including CD4+ T helper cells, CD8+ cytotoxic T cells, NK cells, and NK T cells (35). Tregs rely heavily on OXPHOS and FAO for their energy needs (36). In the TME, where glucose is scarce and lactate is abundant, expression of Foxp3 increases, which in turn suppresses Myc and glycolysis, promotes OXPHOS, and helps Tregs adapt to their environment (37). HIF-1α serves as a metabolic switch in TME, oscillating between glycolysis-driven migration and OXPHOS-driven immunosuppression in Tregs (38). Tregs absorb fatty acids, store them as lipid droplets, and utilize fatty acid synthesis to aid their functional maturation (39). Blocking fatty acid-binding protein 5 in Tregs leads to the release of mitochondrial DNA, which activates cGAS-STING-dependent type I interferon signaling. This process increases the production of the regulatory cytokine IL-10 and suppresses the function of Tregs (40).

TAMs are predominantly of the M2 phenotype and support tumor growth and metastasis by releasing cytokines, chemokines, and growth factors (41). TAMs shift their metabolism towards OXPHOS and FAO while reducing glycolysis and the pentose phosphate pathway in the TME (42). This metabolic reprogramming promotes immunosuppressive signaling, which is favorable for tumor growth in the TME (43). Mast cells induce inflammation by releasing various chemokines and cytokines upon activation by a stimulus, which rapidly degranulates and releases their granule contents into the extracellular space through exocytosis (44). They contain extracellular mitochondrial particles and DNA, which can trigger cytokine release and exacerbate inflammation via autocrine or paracrine signaling (45). Overall, these findings illustrate the complex interplay between mitochondrial metabolic processes and immunosuppressive mechanisms within the TME and highlight potential targets for therapeutic interventions to enhance antitumor immune responses.

## 4 Mitochondrial regulation of cancer cells for immune evasion

Cancer cells exhibit considerable alterations in their metabolic processes, profoundly impacting cancer immunity in TME (**Figure 1**). One of the most prominent mechanisms of metabolic reprogramming is the Warburg effect, wherein glucose uptake and lactate production increase significantly in tumor cells, even in the presence of oxygen (46). Glycolytic activity and elevated glucose uptake by cancer cells can restrict glucose availability to tumor-infiltrating immune cells, thereby promoting immune escape​ (47). Furthermore, increased glycolytic activity in tumor cells correlates positively with elevated PD-L1 expression (48). Lactate, a primary metabolite generated by glycolysis, plays a direct role in facilitating the immune evasion of cancer cells (49). Lactate inhibits the maturation of dendritic cells and impairs their ability to present antigens by downregulating the expression of major histocompatibility complex class II molecules (50). Lactate impairs the effectiveness of cytotoxic T lymphocytes (CTLs) by lowering the production of proinflammatory cytokines, such as IFN-γ, which are essential for CTL-mediated antitumor activity, and by impairing the secretion pathways of cytolytic molecules (51). Tumor-derived lactate reduces NK cell activity by directly inhibiting their cytolytic function, decreasing the expression of activation receptors, such as NKp46, triggering apoptosis in NK cells, and indirectly increasing the number of MDSCs that suppress NK cytotoxicity (32, 52).

Other studies have demonstrated that mitochondrial function positively correlates with the immune evasion ability of cancer cells. In N-acetyltransferase 1-depleted breast cancer cells, a reduction in OXPHOS and mitochondrial biogenesis proteins was observed along with an elevation in antigen presentation proteins (53). The elevated expression of CD147, which is upregulated in various malignant tumors, is associated with increased GLUT1 and MCT1 levels (54). This enhanced glycolytic metabolism in hepatocellular carcinoma (HCC) cell lines correlated with immunosuppressive lymphocyte infiltration in HCC tissues (55). Furthermore, deletion of Complex II, one of the key components of the electron transport chain, inhibits melanoma tumor growth by enhancing antigen presentation and T cell-mediated death (56).

Cancer cells also disrupt mitochondrial metabolism in immune cells. For example, HCC secretes α-fetoprotein, which reduces SREBP-1 and PGC1-α in dendritic cells, leading to lower lipogenesis, oxygen consumption rate, and ATP synthesis of the cell (57). Interestingly, cancer cells can directly hijack mitochondria from immune cells in the TME to evade immune detection and destruction. The transfer of mitochondria from immune cells to cancer cells enhances cancer cell metabolism (58). Furthermore, mitochondria from T cells can transfer to cancer cells, leading to the upregulation of genes involved in cytoskeleton remodeling, energy production, and TNF-α signaling pathways, along with increasing cell cycle activity (59). In conclusion, cancer cells reprogram their metabolism, notably via the Warburg effect, to enhance their growth and evade immune responses. They also disrupt the mitochondrial metabolism and steal mitochondria from immune cells to evade immune detection.

## 5 Targeting mitochondria in cancer immunity

Since mitochondrial regulation plays a key role in cancer immunity, mitochondria-targeting drugs have the potential to boost immune cell function and immunotherapy for cancer. Many drugs targeting mitochondria are being investigated from an immunotherapy perspective, with some currently in clinical trials (**Table 1**).

**Table 1:** Impact of mitochondria-targeting drugs on immunotherapy.

Drugs	Function	Effects on cancer immunity	FDA-approved	Ongoing clinical trials	Selected clinical trials	Purpose
Metformin	Mitochondrial Complex I inhibitor	Protects infiltrating CD8+ T cells from hypoxia-induced immunosuppression, boosts antitumor effects when combined with a PD-1 inhibitor, inhibiting immunosuppressive cells like MDSCs	O	O	NCT03800602	Phase II. Evaluating the effectiveness of metformin with PD-L1 inhibitor nivolumab and metformin in stage IV colorectal cancer
Phenformin	Mitochondrial Complex I inhibitor	Selectively inhibits granulocytic myeloid-derived suppressor cells, boosts antitumor effects when combined with a PD-1 inhibitor Increased infiltration of CD8+ T cells in tumor	X	△	NCT03026517	Phase I. Evaluating the effectiveness of phenformin with BRAF and MEK inhibitor in metastatic melanoma with a BRAF mutation
Canagliflozin	Mitochondrial Complex I inhibitor	Promotes PD-L1 degradation by E3 ligase, suppressing cancer progression by inhibiting the MAPK/ERK and PI3K/AKT pathways	O	△	NCT05090358	Phase II. Evaluating the effectiveness of canagliflozin with alpelisib and fulvestrant in metastatic PIK3CA-mutant breast cancer
IACS-010759	Mitochondrial Complex I inhibitor	Boosts antitumor effects when combined with radiotherapy and anti-PD-1	X	X	–	–
Atovaquone	Mitochondrial Complex III inhibitor	Boosts antitumor effects when combined with a PD-L1 inhibitor	O	△	NCT03568994	Early Phase I. Evaluating the tolerability of atovaquone with chemotherapy in pediatric AML patients
Simvastatin	Mitochondrial Complex III, V inhibitor	Shifts M2 to M1 macrophage, enhancing CD8+ T cell activity and TCR signaling pathway, downregulates PD-L1 expression through the DEPTOR/mTOR pathway	O	△	NCT03324425	Phase II. Evaluating the effectiveness of simvastatin with dual anti-HER2 therapy in metastatic breast cancer
Atorvastatin	Mitochondrial Complex I, III, IV inhibitor	Boosts antitumor effects when combined with a PD-1 inhibitor, inhibits PD-L1 expression through the MAPK pathway	O	△	NCT04862260	Early Phase I. Evaluating the effectiveness of atorvastatin with chemotherapy in solid tumor and acute myeloid leukemia
Hydroxyurea	Mitochondrial oxygen consumption inhibitor	Boosts antitumor effects when combined with a CDK1 inhibitor, impairs differentiation of MDSCs while promoting T cell activation	O	△	NCT03107182	Phase II. Evaluating the effectiveness of hydroxyurea with chemotherapy in head and neck cancer
IR-780	Immunogenic cell death inducer	Enhances dendritic cell maturation and effective T cell priming	X	X	–	–
Venetoclax	Mitochondrial cytochrome c (Cyt C) release inducer	Enhances NK cell function Boosts antitumor effects when combined with a PD-L1 inhibitor	O	O	NCT04277442	Phase I. Evaluating effectiveness of venetoclax with nivolumab and decitabine in TP53-mutated acute myeloid leukemia
Dichloroacetate	Pyruvate dehydrogenase kinases (PDKs) inhibitor	Increases the number of CD8+ T cells and NK cells	X	△	NCT01111097	Phase I. Evaluating effectiveness of dichloroacetate in malignant brain cancer
Bezafibrate	PGC-1α/PPAR agonist	Enhances CD8+ T cell activity Boosts antitumor effects when combined with a PD-L1 inhibitor	X	X	–	–
EnPGC-1	PGC-1α/β Epigenetic activator	Enhances CD8+ T cell activity Boosts antitumor effects when combined with a PD-L1 inhibitor	X	X	–	–

A circle indicates that the drug is currently in clinical trials with immunotherapy. A triangle shows that the drug is currently in clinical trials but not relevant to immunotherapy. A cross means the drug is not undergoing any current clinical trials.

Metformin is one of the most extensively studied mitochondria-targeting drugs that enhances immune responses in cancer therapy, which inhibits Complex I of the mitochondrial respiratory chain (60, 61). Metformin improves cancer immunotherapy by directly protecting tumor-infiltrating CD8+ T cells from hypoxia-induced immunosuppression, potentially by reducing ROS production and preventing apoptosis (62). Combining metformin with an anti-PD-L1 antibody has been shown to induce tumor necrosis by enhancing CD8+ T cell infiltration and increasing IFN-γ expression (63). Furthermore, in STK11 mutant lung cancer, the combination of metformin with a PD-1 inhibitor boosts antitumor effects by inhibiting STING ubiquitination in an AXIN-1-dependent manner (64). Additionally, metformin activates AMPK phosphorylation in various cancer cells, which bolsters antitumor immunity by inhibiting immunosuppressive cells like MDSCs (65, 66). Phenformin, a drug structurally similar to metformin but with a stronger effect on Complex I inhibition, selectively targets MDSCs *in vivo* (67). Furthermore, combining phenformin with anti-PD-1 antibody therapy reduced tumor growth with greater infiltration of CD8+ T cells into a melanoma mouse model (67). Canagliflozin, a medication used to treat type 2 diabetes, is another drug that has been discovered to inhibit mitochondrial complex I activity. On the plasma membrane, SGLT2 (sodium/glucose cotransporter 2) physically interacts with PD-L1, preventing its proteasome-mediated degradation. Canagliflozin disrupts this interaction, leading to PD-L1 degradation by E3 ligase and consequently enhancing the activity of antitumor cytotoxic T cells (68, 69). Additionally, canagliflozin inhibits both the MAPK/ERK and PI3K/AKT signaling pathways, thereby suppressing cancer progression (69). A small molecule that inhibits mitochondrial complex I activity named IACS-010759 decreases radiation-induced Tregs, increases activated CD8+ T cells, and, when combined with radiotherapy and anti-PD-1, promotes abscopal responses and prolongs survival in a non-small cell lung cancer (NSCLC) xenograft mouse model (70). Atovaquone is a mitochondrial complex III inhibitor and alleviates hypoxia in TME in colorectal cancer *in vivo*. Furthermore, combination of atovaquone with anti-PD-L1 antibody greatly enhances tumor eradication in the CT26 colorectal cancer model by establishing a tumor-specific memory immune response (71).

Statins, which inhibit the enzyme HMG-CoA reductase involved in cholesterol synthesis, impair mitochondrial function by inhibiting mitochondrial respiratory chain (72). Seven statin drugs were tested for their ability to inhibit tumor cell proliferation using an *ex vivo* co-culture assay with murine cancer cells and tumor-infiltrating lymphocytes. Among these, simvastatin and lovastatin enhance T cell-mediated tumor cell killing and shift M2 to M1 macrophages in this model (73).

Simvastatin also boosts antitumor immunity by enhancing CD8+ T cell activity and T cell antigen receptor (TCR) signaling pathway (74). Simvastatin also reduced ILF3 expression by lowering H3K14 acetylation levels, and ILF3, in turn, downregulated PD-L1 expression through the DEPTOR/mTOR pathway (75). Another type of statin called atorvastatin significantly enhanced antitumor efficacy by promoting T cell activation in combination with an anti-PD-L1 antibody. Additionally, atorvastatin inhibited the MAPK pathway, leading to decreased PD-L1 expression (76). Hydroxyurea is an FDA-approved drug for treating both sickle cell disease and cancer, and it can be used alone or in combination with conventional chemotherapy or radiation therapy (77). Combining a checkpoint kinase 1 inhibitor with low-dose hydroxyurea reduced the tumor size of melanomas that are resistant to BRAF and immune checkpoint inhibitors *in vivo* (78). Mito-HU, a modified form of hydroxyurea designed to target mitochondria, disturbed differentiation of MDSCs while promoting T cell activation *in vitro* (77). IR-780 boosts cancer immunity by targeting mitochondria to induce immunogenic cell death, which exposes tumor-associated antigens, leading to enhanced dendritic cell maturation, effective T cell priming, and improved immune responses against tumors (79).

NK cells effectively drive cancer cells toward mitochondrial apoptosis, and when combined with the BCL-2 inhibitor venetoclax, they synergistically enhance the killing of cancer cells both *in vitro* and *in vivo* while pre-activated NK cells have been shown to become resistant to venetoclax (80). Furthermore, in combination with immune checkpoint blockade, venetoclax boosts infiltrating effector T cells and strengthens antitumor efficacy (81). Lactate produced by cancer cells promotes the IL-23/IL-17 inflammatory pathway and increases arginase I (ARG1) expression in macrophages, leading to the inhibition of T cell proliferation and activation (82). Dichloroacetate (DCA) reduces the IL-23/IL-17 inflammatory pathway and ARG1 expression in macrophages, while increasing the number of IFN-γ-producing CD8+ T cells and NK cells *in vivo* (82). Bezafibrate, a PGC-1α/PPAR agonist, promotes mitochondrial biogenesis and fatty acid oxidation (FAO) in T cells and increases the accumulation and activation of CD8+ T cells within tumors (83). In a lung carcinoma xenograft model, bezafibrate also enhanced the antitumor effects of PD-1 blockade (83). EnPGC-1, a DNA-based epigenetic activator that induces targeted expression of PGC-1α/β, enhances the mitochondrial activation, energy metabolism, and proliferation of CD8+ T cells *in vitro*. In a mouse model, EnPGC-1 synergizes with PD-1 blockade, leading to enhanced tumor inhibition (84). In summary, research into mitochondria-targeting drugs suggests that these agents can provide synergistic effects when combined with immunotherapy, making them promising candidates for clinical use. However, further investigation is needed to fully understand the underlying mechanisms and optimize their efficacy in clinical settings.

## 6 Conclusion

Despite the emergence of immunotherapy as a new strategy to treat cancer, some cancers are non-responsive to this treatment. Immune checkpoint inhibitors help the immune system recognize and attack cancer cells, but their effectiveness may be limited when used alone due to cancer’s immune evasion mechanisms (85, 86). Even cancer cells that respond to immune checkpoint inhibitors have been reported to develop resistance to immune checkpoint inhibitors over time (87, 88). Mitochondrial-targeted drugs can be used in combination with the treatment to regulate cellular energy metabolism and inhibit the survival and proliferation of cancer cells, thereby reducing the number of cancer cells and creating an environment in which immune cells can function more effectively (65). In addition, changes in the tumor microenvironment can promote the infiltration and activation of immune cells (89). This is expected to further increase the effectiveness of immune checkpoint inhibitors.

Numerous studies already have demonstrated that mitochondrial regulation plays a crucial role in cancer immunity and that mitochondria-targeting drugs enhance the efficiency of immunotherapy. Therefore, targeting mitochondria has the potential to restore and boost immune cell function within the TME and synergistically increase the effectiveness of immunotherapy. However, although many mitochondria-targeting drugs are currently in clinical trial, the primary focus is not directly connected to immunotherapy. In addition, the molecular mechanisms by which mitochondria-targeting drugs enhance the effectiveness of immunotherapy are not yet well understood. Therefore, further research is required to elucidate these mechanisms and their potential as immunotherapy adjuvants.
